# The abundance of epiphytic liverworts on the bark of *Cryptomeria japonica* in relation to different physical and biochemical attributes, found in Senchal Wildlife Sanctuary, Darjeeling, Eastern Himalaya

**DOI:** 10.1186/s12898-019-0253-9

**Published:** 2019-09-11

**Authors:** Sumira Mukhia, Palash Mandal, D. K. Singh, Devendra Singh

**Affiliations:** 1 Plant Physiology and Pharmacognosy Research Laboratory, Department of Botany, North Bengal University, Raja Rammohunpur, District, P.O: NBU, Darjeeling, West Bengal 734 013 India; 2 Botanical Survey of India, CGO Complex, 3rd MSO Building, Salt Lake Sector I, Kolkata, West Bengal 700 064 India

**Keywords:** Epiphytic liverworts, Light, Moisture, Phenols, Terpenes, Abundance

## Abstract

**Background:**

Maintenance of biodiversity is an integral part of sustainable forest management. Epiphytic bryophytes are an important element of biodiversity. Thus, this work aims to study the role of different physical and biochemical factors in affecting the growth and proliferation of epiphytic liverworts. Fifty trees in three different plots, distributed in Senchal wildlife sanctuary, Darjeeling, were surveyed. Factors such as light intensity, moisture, and diameter at breast height (DBH) of the tree were studied to evaluate their possible role in affecting epiphytic liverworts. The effect of bark biochemical characteristics on the abundance of epiphytic liverworts was also studied by undertaking a quantitative test of pH, phenol, flavonoid, ortho-dihydric phenol, terpene, total sugar, and tannin. Multiple regression analysis and principal component analysis (PCA) were carried out to test the effects of these parameters.

**Results:**

Light intensity, moisture, and DBH highly influenced the abundance of liverworts. Old trees had higher epiphytic liverwort cover than younger ones. Bark biochemical properties like pH, phenol, flavonoid, ortho-dihydric phenol, tannin and sugar did not have a significant effect on the epiphytic liverwort cover, while the terpenoid content of the bark reduced liverworts cover.

**Conclusion:**

To sustain the occurrence of epiphytic liverworts in ecosystems, forest management should ensure the presence of old trees. Light intensity and moisture had a large effect on the distribution and abundance of liverworts, so it is important to maintain tree cover, shrub layer, and tree density.

## Background

Bryophytes are an integral part of forest ecosystems and have strong functional relationships with many ecosystem processes [1]. They contribute significantly to forest biomass and photosynthetic production, influence moisture retention and nutrient cycling [2]. Epiphytic bryophytes are considered essential for evaluating forest connectivity and continuity [1]. Bryophytes and lichens are sensitive to changes in microclimatic conditions [3, 4]. Different epiphytic liverwort species have different habitat specificity; some grow in moist shaded habitats and might perish when atmospheric humidity decreases as a result of a loss of forest canopy [5], while others grow on large or old trees [6] and require the maintenance of old trees in forests for their persistence. Availability of suitable substrates, stand age and forest history strongly influences liverwort distribution patterns [7]. Species composition, diversity and their relative frequency in a forest canopy are strongly influenced by the light conditions [8] and moisture level [9]. Such ecological information is therefore essential for sustaining and managing habitats which favour the growth of diverse epiphytic communities in managed stands.

Variation in distribution patterns of epiphytic liverwort communities of similarly aged trees, growing in the same geographical area and under similar climatic condition, suggests the possible involvement of variables other than environmental factors in the distribution and proliferation of epiphytic liverwort species [10]. Epiphyte’s abundance and composition are also strongly related to mineral elements, nutrients, and pH of bark [11]. A variety of plant primary and secondary metabolites are present in the bark. In an in vitro assay, bark extracts exhibited both promoting and inhibitory effects on the growth of lichen ascospore and serodia [12, 13]. Secondary plant phenolics mediate plant responses to pathogenic fungi [14]. Like parasitic fungi, epiphytic liverworts also remain attached to outer cork layers of trees. They have an ecological niche markedly different from plant parasitic pathogen, but still, there are chances that they might get subjected to the same line of defense as parasites. Considering this hypothesis, the study of the effects of bark chemicals is highly essential for sustaining and managing epiphytic communities.

Recently, many studies have been undertaken to examine the factors that limit the species diversity and species composition of epiphytic bryophytes worldwide. However, there is a lack of such studies in Darjeeling although this place harbours a large variety of epiphytic bryophytes. It is a part of the Eastern Himalayan region of India lying between 87º59′ to 88º53′ E and 28º31′ to 27º13′ N. It covers an area of about 2320 km^2^ with altitude ranging between 130 and 3660 meters [15]. Darjeeling has cold and moist climatic conditions which are required for maximum diversity and growth of bryophytes [16]. It is also a major tourist destination. Increasing anthropogenic activities, like deforestation, urbanization, tourism, etc. are posing serious threats to the fauna and flora of this area. Already 10% of the liverworts and hornworts in India are considered as rare and threatened due to various biotic factors [17]. So, the objective of this study is to analyze the effect of different environmental factors on the abundance of epiphytic liverworts. The location selected for the analysis was Senchal wildlife sanctuary, Darjeeling, which is one of the oldest wildlife sanctuaries of India. Located in the lap of Eastern Himalaya, this sanctuary harbours many bryophyte species. The following questions were addressed in this work:

i. How do environmental factors like light intensity and moisture content influence the abundance of epiphytic liverworts?
ii. Is the abundance of epiphytic liverworts affected by the diameter of the tree?
iii. What are the habitat requirements of liverwort species that grows on a tree?
iv. Do tree bark phytochemicals have an allelopathic effect on growth and proliferation of epiphytic liverworts?

## Results

This study demonstrates the effect of different environmental factors and phytochemicals of bark on the relative abundance of the epiphytic liverworts. Liverwort flora on *C. japonica* bark consisted mainly of three species, namely, *Bazzania oshimensis*, *Ptycanthus striatus* and *Pellia epiphylla*, belonging to families Lepidoziaceae, Lejeuneaceae and Pelliaceae, respectively. According to abundance percentage, *B. oshimensis* was the most dominant species while *P. epiphylla* was the rarest.

Stepwise multiple linear regression analysis was carried out to analyse the effect of environmental factors and tree bark phytochemicals on the abundance of epiphytic liverworts. The regression coefficients of each regression model from the stepwise backward simplification model are presented in Table 1. A standardized beta coefficient indicates the strength of the effect of an individual independent variable on the dependent variable. The higher absolute value of the beta coefficient indicates a stronger effect. Model 1, containing all ten predictor variables explained 90.1% of the variation on the abundance of epiphytic liverworts. In model 1, pH of the bark had the highest *p* value, (0.942). The β coefficient of pH content was the lowest in the model, so pH was removed and model 2 was built up with the remaining independent variables. In model 2, ortho-dihydric phenol content had the highest p-value (0.868) and was therefore removed.

**Table 1:** Regression coefficients of models when predicting variables of the abundance of epiphytic liverwort are removed by stepwise backward elimination

Model	Unstandardized coefficients		Standardized coefficients	t	Sig.
	B	Std. error	Beta		
1					
(Constant)	34.458	24.031		1.434	0.160
dbh	0.401	0.127	0.349	3.158	0.003
Phenol content	0.078	0.419	0.020	0.186	0.853
Flavonoid content	0.179	0.744	0.027	0.241	0.811
Orthodihydric phenol content	− 0.330	2.077	− 0.019	− 0.159	0.875
Moisture content	0.910	0.481	0.183	1.892	0.066
pH	− 0.423	5.814	− 0.005	− 0.073	0.942
Light intensity	− 0.002	0.001	− 0.218	− 1.780	0.083
Sugar content	47.689	56.758	0.800	0.840	0.406
Tannin content	− 360.325	431.986	− 0.798	− 0.834	0.409
Terpenoid content	− 2.486	0.704	− 0.332	− 3.533	0.001
2					
(Constant)	33.263	17.307		1.922	0.062
dbh	0.401	0.125	0.350	3.209	0.003
Phenol content	0.077	0.413	0.020	0.186	0.854
Flavonoid content	0.175	0.733	0.026	0.239	0.812
Orthodihydric phenol content	− 0.342	2.045	− 0.019	− 0.167	0.868
Moisture content	0.902	0.460	0.181	1.961	0.057
Light intensity	− 0.002	0.001	− 0.217	− 1.809	0.078
Sugar content	47.614	56.039	0.799	0.850	0.401
Tannin content	− 359.747	426.509	− 0.797	− 0.843	0.404
Terpenoid content	− 2.498	0.675	− 0.334	− 3.702	0.001
3					
(Constant)	32.574	16.607		1.961	0.057
dbh	0.407	0.119	0.355	3.408	0.001
Phenol content	0.048	0.371	0.013	0.129	0.898
Flavonoid content	0.121	0.648	0.018	0.186	0.853
Moisture content	0.895	0.453	0.180	1.978	0.055
Light intensity	− 0.002	0.001	− 0.215	− 1.823	0.076
Sugar content	51.469	50.460	0.863	1.020	0.314
Tannin content	− 389.859	381.957	− 0.864	− 1.021	0.313
Terpenoid content	− 2.473	0.650	− 0.331	− 3.802	0.000
4					
(Constant)	33.125	15.859		2.089	0.043
dbh	0.406	0.118	0.354	3.446	0.001
Flavonoid content	0.173	0.498	0.026	0.348	0.730
Moisture content	0.884	0.439	0.178	2.013	0.051
Light intensity	− 0.002	0.001	− 0.214	− 1.840	0.073
Sugar content	50.255	48.994	0.843	1.026	0.311
Tannin content	− 380.683	370.874	− 0.843	− 1.026	0.311
Terpenoid content	− 2.476	0.642	− 0.331	− 3.854	0.000
5					
(Constant)	35.520	14.139		2.512	0.016
dbh	0.414	0.115	0.361	3.614	0.001
Moisture content	0.862	0.430	0.173	2.004	0.051
Light intensity	− 0.002	0.001	− 0.208	− 1.826	0.075
Sugar content	49.467	48.439	0.830	1.021	0.313
Tannin content	− 378.867	367.027	− 0.839	− 1.032	0.308
Terpenoid content	− 2.498	0.633	− 0.334	− 3.949	0.000
6					
(Constant)	37.107	14.061		2.639	0.011
dbh	0.405	0.114	0.354	3.546	0.001
Moisture content	0.851	0.430	0.171	1.979	0.054
Light intensity	− 0.002	0.001	− 0.225	− 1.996	0.052
Tannin content	− 5.341	30.470	− 0.012	− 0.175	0.862
Terpenoid content	− 2.505	0.633	− 0.335	− 3.959	0.000
7					
(Constant)	36.855	13.836		2.664	0.011
dbh	0.404	0.113	0.352	3.581	0.001
Moisture content	0.856	0.425	0.172	2.016	0.050
Light intensity	− 0.002	0.001	− 0.224	− 2.013	0.050
Terpenoid content	− 2.523	0.618	− 0.337	− 4.084	0.000

Dependent variable: abundance of epiphytic liverworts

Variables of model 3, 4 and 5 explained 90.1% of the variation on the abundance of epiphytic liverworts. In a similar way, sequential stepwise regression eliminated phenol, flavonoid and sugar content from models 3, 4 and 5 since their p-values were 0.868, 0.898 and 0.730, respectively. Thus model 6 was created by eliminating these variables. A total of 89.8% of the variation in epiphytic liverwort abundance was explained by model 6. In this model tannin content had a p-value (0.862) higher than 0.05 so tannin content was removed and model 7 was built. Model 7 consisted of predictor variables—dbh, moisture content, light intensity, and terpenoid content. These variables accounted for 89.8% of the variation in the abundance of epiphytic liverworts. Elimination stopped here since the significance level of predictor variables was either less than 0.05 (dbh = 0.01; terpenoid content = 0) or equal to 0.05 (moisture content = 0.05; light intensity = 0.05). The statistics summary of regression models is presented in Table 2. So according to the results of the logistic regression, the significant portion of the variability in the abundance of epiphytic liverwort is explained by the dbh, light intensity, moisture content and terpenoid content of the tree.

**Table 2:** Statistic summary of regression model

Mode	R	R square	Adjusted R square	Std. error of the estimate	R square change	F change	Sig. F change
1	0.901^a^	0.812	0.764	14.77018	0.812	16.885	0.000
2	0.901^b^	0.812	0.770	14.58537	0.000	0.005	0.942
3	0.901^c^	0.812	0.776	14.41143	0.000	0.028	0.868
4	0.901^d^	0.812	0.781	14.24173	0.000	0.017	0.898
5	0.901^e^	0.812	0.785	14.09540	0.000	0.121	0.730
6	0.898^f^	0.807	0.785	14.10227	− 0.005	1.043	0.313
7	0.898^g^	0.807	0.790	13.94957	0.000	0.031	0.862

Dependent variable: abundance of epiphytic liverworts

^a^Predictors: (Constant), Terpenoid content, pH, Phenol content, Tannin content, dbh, Moisture content, orthodihydric phenol content, Flavonoid content, Light intensity, Sugar content

^b^Predictors: (Constant), Terpenoid content, Phenol content, Tannin content, dbh, Moisture content, orthodihydric phenol content, Flavonoid content, Light intensity, Sugar content

^c^Predictors: (Constant), Terpenoid content, Phenol content, Tannin content, dbh, Moisture content,, Flavonoid content, Light intensity, Sugar content

^d^Predictors: (Constant), Terpenoid content, Tannin content, dbh, Moisture content, Flavonoid content, Light intensity, Sugar content

^e^Predictors: (Constant), Terpenoid content, Tannin content, dbh, Moisture content, Light intensity, Sugar content

^f^Predictors: (Constant), Terpenoid content, Tannin content, dbh, Moisture content, Light intensity

^g^Predictors: (Constant), Terpenoid content, dbh, Moisture content, Light intensity

**Fig. 1:**
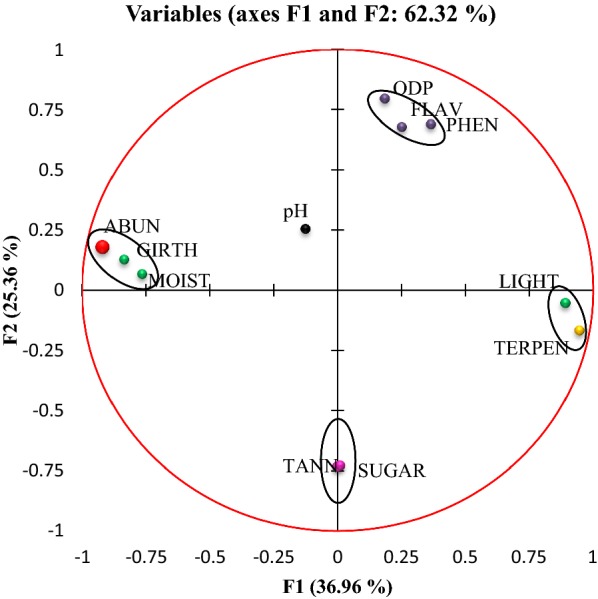
Study of the effect of different physical factors (light, moisture) and biochemical characteristics of plants (pH, girth, phenol, flavonoid, orthodihydric phenol, sugar, tannin and terpenoid content) on the abundance of epiphytic liverwort by Principal Component Analysis. *ABUN* abundance of liverworts, *GIRTH* girth of the tree at breast height, *MOIST* moisture content, *LIGHT* light intensity, *TERPEN* terpenoid content, *ODP* orthodihydric phenol content, *FLAV* flavonoid content, *PHEN* phenol content, *TANN* tannin content, *SUGAR* total sugar content]

For further understanding of the effects of different physical and biochemical factors on the abundance of epiphytic liverworts, Principal Component Analysis (PCA) was performed (Fig. 1). The first two principal components accounted for 62.32% of the data variance. PC1 explained 36.96% and PC2 explained 25.36% of total data variability. Variables were clustered in four groups. The loadings of PC1, abundance, moisture, and girth of the tree were clustered together, showing a positive correlation of these predictor factors (dbh) with the abundance of epiphytic liverworts, while other factors like light and terpenoid were found to be negatively correlated with abundance. The loadings of PC2 were phenol, flavonoid, ortho-dihydric phenol, sugar and tannin. No correlation was found between the loadings of PC2 and the abundance of epiphytic liverworts (Fig. 1). This result further confirmed the involvement of light intensity, moisture content, dbh and terpenoid content on growth and proliferation of epiphytic liverworts.

**Fig. 2:**
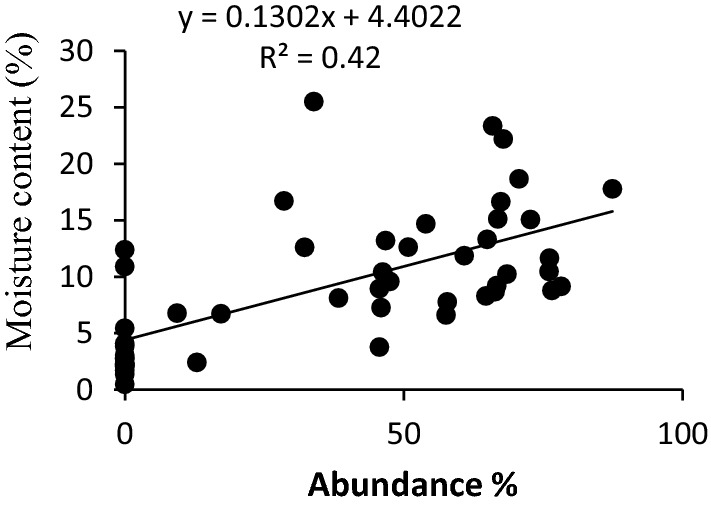
Effect of moisture content on the abundance of epiphytic liverwort

**Fig. 3:**
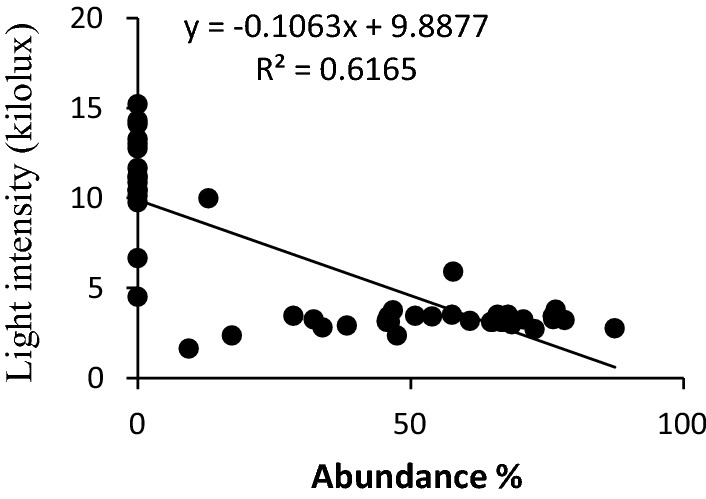
Effect of light intensity on the abundance of epiphytic liverwort

**Fig. 4:**
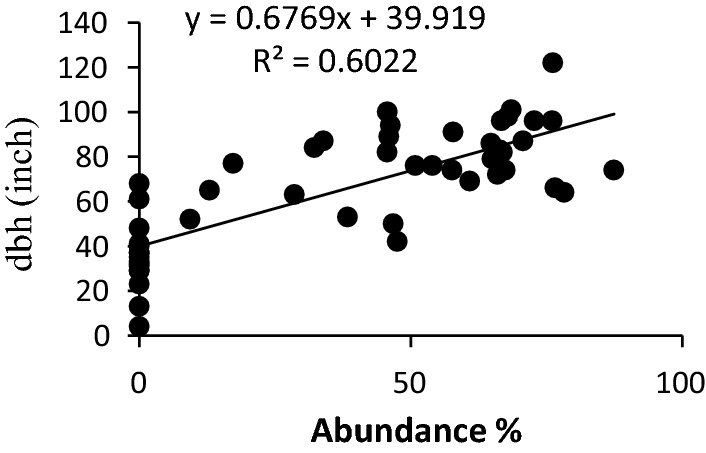
Effect of dbh on the abundance of epiphytic liverwort

**Fig. 5:**
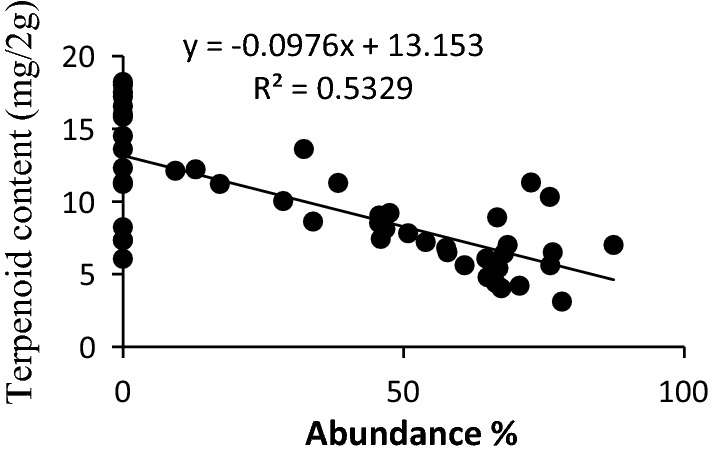
Effect of terpenoid content on the abundance of epiphytic liverwort

The scatter plots for the four significant predictor variables i.e. light intensity, moisture content, dbh, and terpenoid content are presented in Figs. 2, 3, 4, 5. The scatter plot of moisture showed that the abundance of liverwort increased with the increase in the moisture content of the bark (Fig. 2). Barks with moisture content 52.24% to 84.48% favoured luxurious growth of epiphytic liverworts while the frequency of occurrence of liverworts declined drastically below 50% moisture level. Light intensity on the studied sites ranged between 1620 l× to15200 l×. The light intensity of 1900 to 4600 l× was found to be most appropriate for epiphytic liverwort proliferation (Fig. 3). Regression analysis showed a coefficient of determination value of 0.59 between light intensity and the abundance of epiphytic liverwort species. The graph indicated that high light intensity reduces the abundance of epiphytic liverworts.

Presence of epiphytic liverworts was noticed on trees having high dbh, while they were completely absent on trees with low dbh (Fig. 4). Regression analysis showed Coefficients of determination value of 0.635 between the abundance of liverworts and dbh. Epiphytic abundance increased with the increase in the diameter of the tree. Epiphytic liverwort cover was noticed on trees having girth size (dbh) above 42 inches, while no growths were observed on trees having girth size below this point.

Terpenoid is an important class of secondary metabolite synthesized by plants. Terpenoids are the primary constituents of pine oleoresin [18]. Graph suggested that abundance of epiphytic liverwort decreases as the concentration of terpenoid increases in the bark of *C. japonica* tree (Fig. 5).

## Discussion

The study of growth and proliferation of epiphytes on trees occurring in similar environments can be a feasible method for analyzing the effect of physical and biochemical variables on the proliferation of epiphytes. In the present study, the effect of physical factors and bark phytochemicals on the abundance of epiphytic liverwort species in Senchal Wildlife Sanctuary of Darjeeling, Himalaya was studied. Multiple linear regression analysis and Principal component analysis showed epiphytic liverwort abundance in the studied area to be highly correlated with the diameter of a tree at breast height (dbh), moisture content, light intensity and terpenoid content of tree bark. The diameter of the tree exerted a great influence on the distribution of epiphytic liverworts. In the present study, increased abundance of epiphytic liverworts was noticed on trees having large girth. Growth anomaly becomes more frequent and the bark texture becomes more fissured with an increase in dbh which favours the growth of epiphytic liverworts. Similar to this finding, McGee and Kimmere [19], Orjan et al. [7] and Friedel et al. [8] also noticed an increase in epiphytic species abundance with the increase in tree diameter. A positive correlation was also found between dbh and the age of the tree by Lukaszkiewicz and Kosmala [20]. So it seems that large girth sized trees are the most favoured habitat for epiphytic liverwort species. Suitable substrate formation with an increase in tree age might have resulted in the increase in abundance of liverworts. Moreover, the light levels also had a profound influence on the liverwort abundance. Light influences the photosynthetic rate of plants and also the process of transpiration [21], furthermore, it influences the humidity and temperature of the habitat [22]. Most epiphytic liverworts suffer from abrupt exposure to radiation [8, 23]. In the present work, it was found that light levels significantly influenced the growth and proliferation of epiphytic liverwort species. Statistical analysis revealed an interesting pattern of correlation between light intensity and epiphytic liverwort cover. Liverwort abundance declined dramatically above particular threshold limits of light. Luxurious growth of epiphytic liverworts was recorded in light intensity range 1900–4600 lx, above this range density of epiphytic liverworts decreased dramatically. It was also noticed that very low intensity light reduced liverwort abundance. Thus, it is inferred that shade to half shade condition is preferred by the studied epiphytic liverwort species. Moisture is another important physical factor affecting species composition and diversity [24]. Most epiphytic bryophyte species are stenoecious and require shady and highly humid condition [8, 25, 26]. The results of the present study also emphasize the importance of moisture for sustaining the diversity and density of epiphytic liverworts. Statistical analysis suggests that increased moisture content of bark favours luxuriant growth of liverworts. Maximum growth of epiphytic liverworts was noticed when the moisture content of the substrate was more than 50%.

Biochemical characteristics of bark could also affect epiphytes [27]. The probable effect of plant metabolites on the proliferation of epiphytic liverwort was studied. A correlation between richness of epiphytes and the pH of tree bark has been recorded by various authors [28, 29], however, in our study variation in epiphytic liverwort cover was not significantly affected by the pH of the bark. The tree *C. japonica* exudes resin, the major components of which are terpenes. The resin protects plants from invading pathogens [30]. We therefore studied the possible effect of terpenes on epiphytic abundance. We have noticed an increase in liverwort abundance when the terpenoid content of tree bark decreased. This result, therefore, suggests that the phytochemical content of the bark has some allelopathic effect on epiphytic liverworts, even though, unlike pathogens, bryophytes do not invade the sieve or bast tissues of the tree but remain attached to cork layers through rhizoids. Kim [31] has noticed a negative correlation between terpenes and age of the tree. Therefore it would be erroneous to say that reduced terpenoid content alone is responsible for increased abundance of epiphytic liverworts. Thus it can be interpreted that combined effect of decreased terpenoid content and increased dbh might have resulted in luxuriant growth of epiphytic liverworts on large girth sized trees. However, more detailed analysis is required in this field.

Phenolics are mostly involved in plant defense. However, in the present work, the phenolic compounds (phenol, flavonoids, orthodihydric phenol, and tannin) and sugar content didn’t have allelopathic effects on epiphytic liverwort abundance. Epiphyte abundance varied irrespective of the phenolic content of the bark. Since the site used for the present research is an unmanaged forest it is difficult to determine the exact age of trees. Trees under study were categorized as young when dbh < 130 cm, moderate when 130 < dbh < 230 cm and old when dbh > 230 cm. No correlation was observed between the dbh of the tree and phenolic compounds in the present study. Different environmental stresses experienced by the trees might have contributed to variation in the phenolic content of trees undertaken for the present study.

From the statistical analysis, it can be concluded that high moisture content, as well as a suitable substrate, is required to ensure the occurrence and luxuriant growth of epiphytes. Moreover, tree density also affects the density of liverworts, as thinning of the forest after logging leads to exposure of species to radiation [32]. Abrupt exposure to light reduces the abundance of species requiring consistent humidity and shady condition [8]. High light intensity is also detrimental for moisture-loving epiphytic species. It is clear from this study that old growth forest stands containing large old trees, dead logs and canopy cover preventing exposure to sunlight is important for epiphytic growth and conservation. With an increase in the diameter of the trees, the physical and chemical characteristics of bark changes continuously [33]. The trees become more porous and absorbent facilitating the settlement of epiphytic species [34]. Results of the study also suggest that an increase in tree girth benefits epiphytes not only because it increases the amount of substrate available but also because it is associated with reduced terpene content in older trees.

## Conclusion

Senchal Wildlife Sanctuary is one of the important tourist destinations in Darjeeling. Increasing human activities are posing a serious threat to the biodiversity of this area and this increases the chances of the disappearance of some sensitive plant species from this area without even being noticed. Bryophytes and lichens are highly sensitive to small changes in the microclimatic condition of their habitat and this characteristic increases the chances of loss of these species. Bryophytes have always been neglected from the ecological as well as biochemical point of view, as compared to other plant groups. This study showed that the diameter of the tree at breast height (dbh) is the key factor influencing the abundance of epiphytic liverworts. Increased dbh resulted in luxuriant growth of liverworts, suggesting old trees are the preferred habitat of epiphytic liverworts. Terpenoid content of the tree also influenced the epiphytic liverwort abundance. Moreover, moisture content and light condition were found to be of great importance for the proliferation of epiphytic liverworts. So, care should be taken to maintain the tree density and canopy cover as abrupt exposure to light may prove lethal for epiphytic liverwort diversity and density. Thus, forest management, or the responsible authority, should ensure the presence of old, large and rough-barked trees to sustain the epiphytic liverwort cover in this area.

## Methods

The area selected for the study of epiphytic liverworts is Senchal Wildlife Sanctuary (set up in 1915) situated in the Darjeeling District of West Bengal, India (87º59′ to 88º53′E and 28º31′ to 27º13′N). It is one of the oldest wildlife sanctuaries of India. Its elevation ranges between 1500 to 2600 m and covers an area of 38.88 km^2^. The mean annual precipitation in the area is 2981.8 mm, while the mean annual temperature varies between 8.9° and 15.98 °C. The present study was conducted in the year 2016 during monsoon season *i.e*. June to October. As *Cryptomeria japonica* favours luxuriant growth of epiphytic liverworts and also is a dominant tree species in this sanctuary, the study of abundance of epiphytic liverworts was conducted on a total of fifty *C. japonica* trees of different age groups in three plots in forest stands. On the basis of the diameter at breast height (dbh), studied trees were grouped into three categories, viz. young (dbh < 130 cm), moderate (130 < dbh < 230 cm) and old (dbh > 230 cm). Studied sample plots were selected randomly in an area representative of the whole forest stand. Three sample plots of 15 m × 15 m were established. Sample plots were 50 m apart from each other. Epiphytic liverwort abundance was studied on 16 to 17 trees in each plot. In each plot, trees were selected based on the size of the girth of the tree. Equal number of young (dbh < 130 cm), moderate (130 < dbh < 230 cm) and old aged (dbh > 230 cm) trees were studied in each plot. Density and presence or absence of the epiphytic liverworts was recorded up to diameter at breast height (dbh). On each tree, five quadrates of 5 cm × 5 cm size were placed randomly up to diameter at breast height for the sampling of bryophytes along the underlying bark [9]. Effect of moisture content, light intensity, dbh and biochemical attributes of bark on the abundance of epiphytic liverworts were studied in this work.

### pH measurement

The method described by Mezaka et al. [35] was followed for pH measurement. At first, all the epiphytic species (including mosses, lichens, epiphytic liverworts) covering the bark were removed. Tree bark (0.5 g) was cut into small pieces and added to 20 ml 1 M KCl solution and shaken for 1 h. After 1 h pH was measured using pH meter (Digital pH meter model no. 335, Systronics, India).

### Bark extract preparation

Bark extract was prepared by refluxing 3 g powdered bark in methanol for 2 h. The extracts were then filtered and used for estimating phenol, flavonoid, ortho-dihydric phenol, tannin, and sugar content of tree bark.

### Phenol estimation

Phenol content of the bark was estimated following the method of Kadam et al. [36]. Methanolic extract (1 ml), 1 ml of 95% ethanol, 5 ml of distilled water and 0.5 ml of 50% Folin–Ciocalteau reagent was mixed. 1 ml of 5% Na_2_CO_3_ was added to the entire mixture after 5 min. The absorbance was measured at 725 nm using UV–VIS Double Beam Spectrophotometer 2201, Systronics. The standard curve was calibrated using different concentrations of gallic acid.

### Flavonoid estimation

Flavonoid content was estimated following the method of Atanassova et al. [37]. Bark extract (0.5 ml) was mixed with 4 ml of distilled water and 0.3 ml of 5% NaNO_2_. After 5 min, 0.3 ml of 10% AlCl_3_, and 2 ml of 1.0 M NaOH were added. The whole mixture was diluted by adding 2.4 ml of distilled water. Absorbance was measured at 510 nm. Quercetin was used as standard.

### Orthodihydric phenol estimation

The Method described by Mahadevan and Sridhar [38] was followed. Bark extract (0.5 ml), 0.5 ml of Arnow’s reagent, 5 ml of H_2_O and 1 ml of 1(N) NaOH were mixed and absorbance was measured at 515 nm. Catechol was used as standard.

### Tannin estimation

The method described by Thimmaiah [39] was followed for tannin estimation. The reaction was initiated by mixing 1 ml of extract, 5 ml of water, 0.5 ml of Folin-Denis reagent and 1 of ml 1 M sodium carbonate. Absorbance was measured at 700 nm after 30 min of incubation. Tannic acid was used as standard.

### Terpenoid estimation

The method described by Theng and Korpenwar [40] was followed. Bark powder (2 g) was soaked in 50 ml of 95% ethanol for 24 h. It was then filtered; the filtrate was extracted with petroleum ether (60–80 °C). After extraction, the petroleum ether fraction was kept and dried. Content of total terpenoid was determined from the extractive weight of the petroleum ether fraction by gravimetric method.

### Total soluble sugar estimation

Bark powder (100 mg) was boiled for 3 h with 5 ml of 2.5 N HCl in a water bath. The extract was neutralized by adding sodium carbonate and the total volume was made up to 100 ml. The mixture was centrifuged and the supernatant was used for estimating total soluble sugar following the earlier described method of Thimmaiah [39]. Bark extract (1 ml) was mixed with 4 ml Anthrone reagent and heated for 8 min in a boiling water bath. The reactant was cooled rapidly and the absorbance was measured at 630 nm.

### Moisture content

Tree bark (3 g) was dried at 50 °C for 1 day until it lost all the moisture and its weight was stabilized. Bark was weighed again. Moisture content was measured using the following formula

$${\text{Moisture \% }} = \frac{{{\text{Initial weight}} - {\text{Weight after drying}}}}{{ {\text{Initial weight}}}} \times 100$$

### Light intensity

The intensity of light falling on a particular area was measured using Lutron lux meter LX-101.

### Data analysis

A multiple Linear Regression Analysis was run using SPSS (Version 12.00) to identify the key predictor variables affecting epiphytic liverwort abundance. In the present work, the linear relationship between one dependent variable (abundance of epiphytic liverworts) and ten independent variables (dbh, moisture content, light intensity, pH, phenol, flavonoid, ortho-dihydric phenol, tannin, terpenoid, and sugar content) was studied. Backward elimination was used to identify the most important variables affecting the abundance of epiphytic liverworts. First, all independent variables were included in the regression and their significance was assessed. The independent variable with the lowest contribution to the regression equation (highest p-value) was removed. This elimination procedure was repeated with the remaining variables and continued until only variables with significant effects remained in the regression.

Principal Component Analysis (PCA) using Multivariate Statistical Package (MVSP 3.1) was also used to explore relationships of physical and biochemical attributes with the abundance of epiphytic liverworts. The scatter plot showing the relation between important predictor variables and liverwort abundance was prepared using MS Excel 2007 (Microsoft, Redmond, WA, USA).

## Data Availability

All data generated or analyzed during this study are included in the manuscript and the raw data will be made available on request.

## Abbreviations

PCA: principal component analysis
dbh: diameter at breast height
ABUN: abundance of liverworts
GIRTH: girth of the tree at breast height
MOIST: moisture content
LIGHT: light intensity
TERPEN: terpenoid content
ODP: orthodihydric phenol content
FLAV: flavonoid content
PHEN: phenol content
TANN: tannin content
SUGAR: total sugar content
